# Dissociating sub-processes of aftereffects of completed intentions and costs to the ongoing task in prospective memory: A mouse-tracking approach

**DOI:** 10.3758/s13421-022-01289-z

**Published:** 2022-02-25

**Authors:** Marcel Kurtz, Stefan Scherbaum, Moritz Walser, Philipp Kanske, Marcus Möschl

**Affiliations:** 1 Department of Psychology, Technische Universität Dresden, 01062 Dresden, Germany; 2 Max Planck Institute for Human Cognitive and Brain Sciences, Leipzig, Germany

**Keywords:** Prospective memory, Intention deactivation, Commission errors, Mouse tracking, Delay theory, Monitoring, Costs, Spontaneous retrieval

## Abstract

In the present study, we used mouse tracking to investigate two processes underlying prospective memory (PM) retrieval: First, we aimed to explore to what extent spontaneous retrieval of already completed PM intentions is supported by reflexive-associative and discrepancy-plus-search processes. Second, we aimed to disentangle whether costs to an ongoing task during the pursuit of a PM intention are associated with presumably resource-demanding monitoring processes or with a presumably resource-sparing strategic delay of ongoing-task responses. Our third aim was to explore the interaction of processes underlying costs to the ongoing task and processes of spontaneous retrieval. Our analyses replicated response-time patterns from previous studies indicating aftereffects of completed intentions and costs to ongoing-task performance, as well as increased aftereffects while pursuing a PM intention. Notably, based on our mouse-tracking analyses, we argue that aftereffects of completed intentions are best explained by a reflexive initiation of an already completed intention. If the completed intention is not performed in its entirety (i.e., no commission error), the reflexive initiation of the completed intention is followed by a subsequent movement correction that most likely represents a time-consuming response-verification process. Regarding performance costs in the ongoing task, our analyses suggest that actively pursuing a PM intention most likely leads to a strategic delay of ongoing activities. Lastly, we found that pursuing a novel PM task after intention completion exacerbated orienting responses to all deviant stimuli, exacerbated the readiness to initiate the completed intention reflexively, and substantially prolonged the response-verification process following this reflexive intention retrieval.

## Introduction

The term prospective memory (PM) subsumes abilities necessary for maintaining an intention in memory and retrieving it in the future (Cohen & Hicks, 2017; Rummel & McDaniel, 2019). These abilities enable goal-directed behavior in everyday life, such as putting a letter in a mailbox while we are on our way to work. In this example, having the intention to put a letter in a mailbox would likely prompt us to look for mailboxes on the side of the road and might also slow us down on our way to the office – our ongoing task in this case. After we have mailed the letter, we have completed our intention and no longer need to look out for any mailboxes. This deactivation of completed intentions is considered to be essential for goal-directed behavior, given that it enables focusing on new task demands and might prevent perseverative behavior (Goschke & Bolte, 2018; Mayr & Keele, 2000). Previous studies, however, showed that completed intentions can interfere with subsequent tasks in terms of aftereffects of completed intentions (Möschl et al., 2020). What are the mechanisms behind such aftereffects, and what causes us to slow down our ongoing-task performance while pursuing an intention in the first place? While aftereffects of completed intentions have been suggested to occur due to continued spontaneous retrieval of intentions after their completion, little is known about the underlying retrieval processes (Bugg & Streeper, 2019). Similarly, while several studies consistently reported costs to an ongoing task while actively pursuing a PM intention, the mechanisms underlying these costs are still a subject of discussion (Anderson et al., 2019). Therefore, in the present study, we aimed to elucidate further the mechanisms underlying spontaneous retrieval of completed intentions and costs to the ongoing task using mouse tracking as a continuous behavioral measure.

### Spontaneous retrieval of completed intentions

Over the past years, PM research investigated the deactivation of completed intentions within event-based PM paradigms with no-longer-relevant PM cues (Anderson & Einstein, 2017; Bugg et al., 2013; Bugg et al., 2016; Möschl et al., 2020; Walser et al., 2012; Walser et al., 2017). In these paradigms, participants first perform an event-based PM task, in which they are instructed to perform a particular action (PM response, e.g., press spacebar) once they notice a specific PM cue that signals an opportunity to retrieve and execute the intended action (e.g., a specific symbol). The PM task is embedded in an ongoing task (e.g., digit categorization), which occupies participants’ attention. At the end of this so-called *active phase* of the task, participants are instructed that the PM task is finished and that they will continue performing the ongoing task in the subsequent *finished phase*. To assess aftereffects of the completed intention, PM cues of the former (now finished) PM task are presented as so-called PM_REPEATED_ cues to participants during the finished phase. Participants are instructed to ignore the PM_REPEATED_ cue in these trials and respond to the ongoing task instead. Additionally, to provide a baseline for assessing orienting responses to deviant stimuli, in some trials, control cues (so-called oddball cues) that never served as PM cues during the experiment are presented to participants. A key finding of studies investigating intention deactivation in these paradigms is that participants’ ongoing-task responses are slower in PM_REPEATED_ trials than in oddball trials, indicating aftereffects of completed intentions (Beck et al., 2014; Walser et al., 2012; Walser et al., 2017). Additionally, in some cases, participants actually perform the no-longer-relevant PM response in PM_REPEATED_ trials (so-called commission errors) (e.g., Anderson & Einstein, 2017; Scullin et al., 2012; Walser et al., 2017).

Aftereffects of completed intentions and commission errors have been explained by the persistence of spontaneous retrieval of a completed intention in response to PM_REPEATED_ cues (Anderson & Einstein, 2017; Scullin et al., 2012; Scullin & Bugg, 2013; Walser et al., 2012). Additionally, the dual-mechanisms account of PM commission errors states that after a completed intention is spontaneously retrieved, time-consuming cognitive control processes are recruited to prevent the execution of the no-longer-relevant PM response, and commission errors occur when cognitive control is impaired or fails (Bugg et al., 2016; Bugg & Scullin, 2013; Matos et al., 2020; Scullin & Bugg, 2013).

The term *spontaneous retrieval* refers to retrieval processes that do not require volitional processes to detect a PM cue (McDaniel & Einstein, 2000, 2007). While spontaneous intention retrieval has been likened to the experience of an intention "popping into mind" (Einstein et al., 2005; Meier et al., 2006), it does not equate to automatic intention fulfillment. Instead, after an intention has been retrieved spontaneously, further top-down processes are necessary to maintain and fulfill the intention (Einstein et al., 2003; Shelton et al., 2019). Notably, according to the (dynamic) multiprocess framework of PM, at least two sub-processes could account for the spontaneous retrieval of active or completed intentions: a reflexive-associative process and a discrepancy-plus-search process (McDaniel et al., 2004; McDaniel et al., 2015; McDaniel & Einstein, 2000; Scullin et al., 2013; Shelton et al., 2019). Both of these processes can account for aftereffects of completed intentions, and both are possible mechanisms underlying spontaneous intention retrieval within the dual-mechanisms account of PM commission errors. The reflexive-associative processes view posits that after an intention is stored in long-term memory, complete processing of a PM cue leads to a high probability of *automatic bottom-up* retrieval of the associated intended action (Loft & Yeo, 2007; McDaniel et al., 1998; McDaniel et al., 2004). By contrast, the discrepancy-plus-search-process view posits that processing a PM cue during an ongoing task causes an experience of a discrepancy, which triggers a search process in memory regarding the source of the experienced discrepancy. This discrepancy-plus-search process then may result in the retrieval of the intended action only after an effortful *controlled top-down* search in memory (Breneiser & McDaniel, 2006; Lee & McDaniel, 2013; McDaniel & Einstein, 2007).

It is unknown to what extent and under what conditions reflexive-associative and discrepancy-plus-search processes are involved in the genesis of aftereffects of completed intentions. Similarly, it remains unclear at which point or when cognitive control becomes involved in intention deactivation and thus contributes to RT aftereffects. Does cognitive control inhibit the retrieval of an intention itself, does it only inhibit its execution, or both?

To gain insight into these issues, in our view, it is necessary to specify and extend PM theories with specific assumptions and predictions regarding the microstructure or precise sequences of (spontaneous) intention retrieval processes and cognitive control engagement in PM. Additionally, it requires utilizing research methods capable of mapping these processes into discrete sequences to test these predictions. As a starting point, specifying subprocesses of spontaneous retrieval of completed intentions could inform research on intention deactivation in at least two ways. First, it could help to clarify whether and when intention deactivation involves disassembling the memory representation of the PM cue as the target of an intention (discrepancy-plus-search process) or destabilizing the cue-response association between PM cue and PM response (reflexive-associative process), as has recently been suggested by Shelton et al. (2019). Second, it could aid in specifying the target(s) of cognitive control processes within the dual-mechanisms account of PM commission errors. If completed intentions were retrieved via a discrepancy-plus-search process, cognitive control processes could presumably already inhibit or interrupt the memory search, which would prevent full retrieval of the completed intention (early inhibition) (but see Anderson & Einstein, 2017). By contrast, if completed intentions were retrieved via reflexive-associative processes, cognitive control processes could presumably only inhibit executing the intention after it had been retrieved (late inhibition).

Notably, since both spontaneous retrieval processes could manifest in slowed responses and/or commission errors (e.g., Bugg & Streeper, 2019; Möschl et al., 2020), neither of these subprocesses can be distinguished directly by discrete measures like RT aftereffects or commission errors alone. Instead, however, they should become distinguishable in continuous behavioral measures that allow an investigation of the genesis of a reflexively triggered response over time. Therefore, the present study's first aim was to investigate whether aftereffects of completed intentions are caused by a reflexive-associative process or a discrepancy-plus-search process.

### Costs to the ongoing task

Our study's second aim was to disentangle the processes underlying costs to the ongoing-task performance when participants perform a PM task. These ongoing-task costs are typically assessed by comparing ongoing-task performance between conditions in which participants additionally perform a PM task and conditions in which participants perform only an ongoing task (i.e., ongoing-task-only conditions). The term ongoing-task costs refers to findings of slower and sometimes more error-prone ongoing-task performance when participants maintain an intention of a PM task (Einstein & McDaniel, 2010; McDaniel & Einstein, 2000; Smith, 2003, 2010; Smith et al., 2007; Smith & Bayen, 2004). These ongoing-task costs were interpreted as evidence that intention retrieval in some PM tasks does not occur spontaneously but involves top-down controlled processes (Guynn, 2003; McDaniel & Einstein, 2000; Smith, 2003). Two theories have recently proposed different processes underlying costs to the ongoing task: capacity sharing theories and the delay theory.

According to capacity-sharing theories of PM, ongoing-task costs result from monitoring processes that draw on limited top-down attentional resources shared between the ongoing task and PM tasks and are presumably required to maintain an intention in memory and search the environment for PM cues (Guynn, 2003; McDaniel & Einstein, 2000; Smith, 2003). Corroborating this assumption, PM accuracy was reported to worsen under increased attentional requirements (i.e., increased capacity sharing) by the ongoing task, which presumably decreased available resources for performing the PM task (Einstein et al., 1997). Further, Brewer et al. (2010) found that ongoing-task cost and PM accuracy were mediated by working memory capacity, suggesting that limited resources are required for both the ongoing and the PM task.

By contrast, according to the delay theory of PM, ongoing-task costs during PM tasks are not the result of shared capacity but instead represent a strategic slowing of ongoing-task responses to allow evidence accumulation about PM cues before making a response (Heathcote et al., 2015; Loft & Remington, 2013; Strickland et al., 2017). This accumulation of information enables the detection of PM cues before an ongoing-task response is made. Consequently, the delay theory posits that during the pursuit of an intention, ongoing-task performance should become slower and more accurate (i.e., more careful responding). Loft and Remington (2013) provided support for the delay theory by showing that increasing time for evidence accumulation about PM cues by introducing relatively short forced delays between stimulus presentation and response opportunity (400–1,600 ms in their study) reduced ongoing-task costs and improved PM accuracy compared to shorter (200 ms) or no-delay conditions.

Using response-time (RT) data and standard significance tests, it is difficult to determine whether ongoing-task costs are caused by a strategic delay or capacity sharing because both approaches predict slowed ongoing-task responses. For this reason, several studies used evidence accumulation models to investigate processes underlying PM and ongoing-task costs (Boywitt & Rummel, 2012; Heathcote et al., 2015; Horn et al., 2011; Horn & Bayen, 2015; Strickland et al., 2017). In evidence accumulation models, the distribution of RTs of a decision process (e.g., an ongoing task response) is modeled by several parameters (Ratcliff & McKoon, 2008), of which drift rate, response threshold, and non-decision time are the most important in the majority of studies. Previous research has used changes in these parameters under different conditions to infer cognitive processes (e.g., Fudenberg et al., 2020; Johnson et al., 2017; Pedersen et al., 2017; Ratcliff et al., 2016). In studies using evidence accumulation models, drift rate is usually associated with information processing speed, while response threshold is related to the response criterion, in other words, how much information needs to be gathered before a decision is made. The non-decision-time parameter represents processes unrelated to the accumulation process, such as response production (Voss et al., 2004).

After several studies showed that capacity-sharing processes and strategic delays could be mapped to specific changes in these model parameters (Boywitt & Rummel, 2012; Horn et al., 2011; Horn & Bayen, 2015), Heathcote et al. (2015) used evidence-accumulation models of ongoing-task response times to examine explicitly whether capacity-sharing processes or a strategic delay caused costs to the ongoing task (see also Strickland et al., 2017).^1^ Heathcote and colleagues argued that in conditions with a PM task compared to conditions without a PM task, capacity-sharing processes are reflected in a slower drift rate, while a strategic delay is reflected in a higher response threshold. According to Heathcote et al. (2015), changes in the non-decision time parameter are less evident concerning capacity-sharing and strategic delay; both processes could be associated with this parameter.

Despite this clear theoretical assignment of capacity-sharing and delay processes to parameters in evidence accumulation models, findings on the effects of a PM task on these parameters are inconsistent. That is, while some studies found that performing a PM task only altered the threshold parameter, which is consistent with the delay theory (Heathcote et al., 2015; Strickland et al., 2017), other studies found changes in all parameters (Boywitt & Rummel, 2012; Horn et al., 2011). Adding to these inconsistencies, Anderson et al. (2018) recently found evidence for both capacity-sharing and delay processes when manipulating task performance strategies in a PM paradigm. In their study, participants were instructed to either use a delayed-responding strategy, a monitoring strategy (experimental instructions), or received no specific instruction (standard condition). Consistent with the delay theory, the delay instruction resulted in a higher response threshold. At the same time, however, Anderson and colleagues also found that participants in the standard PM condition were more likely to use a monitoring strategy than a delay strategy.

In summary, modeling PM-task response times and error rates with evidence accumulation models provides interesting insights into the processes that may underly ongoing-task costs. However, even these modeling approaches have not conclusively clarified whether costs to the ongoing task are due to capacity sharing or a strategic delay. One reason for this is disagreement among authors about the extent to which the processes that underly costs can be represented in the parameters of evidence accumulation models and the fact that several studies provide evidence for both explanations of costs to the ongoing task (Anderson et al., 2019). Advancing the debate between these two views might require new research paradigms. Therefore, our study's second aim was to assess the underlying processes of ongoing-task costs by using a continuous response measure.

### Relationship between cue properties, spontaneous retrieval, costs to the ongoing task, and new PM task sets

What influences whether we base remembering an intention on processes that entail costs to the ongoing task or rely on spontaneous retrieval? Recent research revealed that specific properties of PM cues and PM tasks affect ongoing-task costs as well as the probability of spontaneous retrieval of active and completed intentions (Bugg & Streeper, 2019; Einstein et al., 2005; Einstein & McDaniel, 2005, 2010; Möschl et al., 2020). Two crucial factors are the perceptual salience of the PM cue and its focality, which represents the degree of processing overlap between detecting a PM cue and performing the ongoing task (; Scullin et al., 2010). Focal PM cues exhibit a high processing overlap with the ongoing task. For example, identifying the PM cue "fish" during an ongoing lexical decision requires the same operations needed to make a word versus non-word decision for the ongoing task. By contrast, non-focal PM cues exhibit a low processing overlap with the ongoing task, so additional processes are required to identify a PM cue. For example, identifying the syllable "tor" during a lexical decision task requires scanning the syllables of a stimulus in addition to reading the stimulus. While focal PM tasks often incur minor to no ongoing-task costs and presumably foster spontaneous intention retrieval, particularly when the PM cues are perceptually salient, non-focal PM tasks, and to a lesser extent nonsalient PM cues, often incur substantial ongoing-task costs and intention retrieval is presumed to rely more on top-down controlled retrieval processes (Einstein et al., 2005; Einstein & McDaniel, 2010; Scullin et al., 2010).

In addition to these factors, performing another PM task after intention completion can increase the probability of erroneously spontaneously retrieving a completed intention.^2^ This effect has been shown, for instance, in terms of increased RT aftereffects and higher commission-error rates when another PM task is performed during the finished phase of a repeated PM cue paradigm (e.g., Anderson & Einstein, 2017; Walser et al., 2017). On an abstract level, one potential explanation for this effect is that encoding a new intention after intention completion could establish a general PM-task set or retrieval mode (Guynn, 2003; Underwood et al., 2015) that increases sensitivity to any type of deviant cues in the task. This heightened sensitivity presumably increases the probability that PM_REPEATED_ cues are detected, and a completed intention is retrieved (Möschl et al., 2020; Walser et al., 2017).

Specifying the processes underlying spontaneous intention retrieval and ongoing-task costs and their potential interaction(s) would help shed light on this issue, as several additional explanations for this effect are feasible. First, a strategic delay of ongoing task responses that allows accumulation of evidence for cues of the new PM task could also increase evidence accumulation for PM_REPEATED_ cues, which in turn would increase the probability that the completed PM task is retrieved. Similarly, resource-demanding monitoring for PM cues of a new intention could increase the probability that a PM_REPEATED_ cue is detected, increasing the probability that the completed PM task is retrieved. Second, when considering different subprocesses of spontaneous intention retrieval, it is feasible that performing a PM task during the finished phase leads to stronger experiences of discrepancy and/or a prolonged search in memory for the relevance of PM_REPEATED_ cues, which would exacerbate RT aftereffects. Third, it could also lead to source confusion or source-monitoring errors through which a completed PM task could be confused with a currently active PM task upon encountering a PM_REPEATED_ cue. These effects could, for instance, explain findings of increased PM-related thoughts following PM_REPEATED_ cues (Anderson & Einstein, 2017) or findings of commission errors with the currently active PM response instead of the no-longer-relevant PM response (Walser et al., 2017). Lastly, when considering the dual-mechanisms account of PM commission errors, it is feasible that resource-demanding monitoring processes after intention completion reduce cognitive resources to mobilize cognitive control that may be needed to inhibit retrieval or performance of a completed intention or to inhibit a memory search for the relevance of an experienced discrepancy when encountering a PM_REPEATED_ cue.

Taken together, these speculations illustrate that there are many ways through which performing a PM task after intention completion could exacerbate aftereffects and affect intention deactivation. However, it is difficult to test these predictions and assess their validity without more detailed knowledge about the processes underlying ongoing-task costs and spontaneous retrieval of completed intentions and proper methods to assess them. We are confident that the time-continuous measures of mouse-movement data in our paradigm provide viable tools for this, as they allow a more detailed description of processes underlying ongoing-task costs and spontaneous intention retrieval. Therefore, our study's third aim was to replicate our previous findings of increased aftereffects of completed intentions when participants engaged in a novel PM task during the finished phase (Walser et al., 2017) and elucidate how performing another PM task after intention completion affects intention deactivation and sub-processes of spontaneous retrieval of completed intentions.

### The present study

In summary, with paradigms commonly used to study aftereffects of completed intentions, it is difficult to disentangle the effects of spontaneous retrieval and cognitive control in isolation (Bugg & Streeper, 2019). Furthermore, as we detailed in the previous sections, it is challenging to distinguish current PM theories from one another when using only discrete measures of error rates and RT commonly used in PM research. Problems arise because the juxtaposed processes and theories can affect these measures in the same way. For example, both retrieval of a completed intention through a reflexive-associative process and retrieval through a discrepancy-plus-search process would lead to RT aftereffects and/or commission errors. Similarly, both strategic delay and capacity-sharing processes would be reflected in costs to the ongoing task. Consequently, recent PM research has highlighted the need for new measures other than ongoing task costs that can be used to distinguish between separate PM processes (Shelton et al., 2019). In the following, we elaborate on the time-continuous measures of mouse tracking that we believe can constitute such a measure.

The present study had three goals: First, we aimed to elucidate the extent to which aftereffects of completed intentions arise from a reflexive-associative process or a discrepancy-plus-search process. Second, we aimed to test whether costs to the ongoing task are caused by capacity sharing processes or a strategic delay. Third, we investigated the influence of a new PM task on the effects of spontaneous retrieval. We used fine-grained, time-continuous analyses of mouse-tracking data to achieve these aims.

#### Mouse tracking

In mouse-tracking paradigms, participants usually respond by moving a mouse cursor from a start box at the lower edge of the screen into different response boxes at the upper-left or upper-right corner of the screen. The position of the cursor on the screen is continuously tracked and analyzed. In addition to information about the endpoints of the response selection process, like in simple binary-choice RT or error analyses, mouse tracking allows us to investigate the continuous genesis of response selection in real-time (J. B. Freeman et al., 2011). Continuous response data provides information about cognitive processes that may occur during response selection and affect the curvature of a mouse movement: The strength of the deviation from an ideal straight-line movement into a response box. A more significant curvature has been attributed in previous studies mainly, but not exclusively, to response conflicts (Calcagnì et al., 2017; Kieslich & Hilbig, 2014; Scherbaum et al., 2010; Spivey et al., 2005; Spivey & Dale, 2006). The stronger a movement deviates from an ideal straight line in the direction of an alternative response box, the more conflict occurs in the response selection process. Recently, Erb (2018; see also Fischer & Hartmann, 2014) suggested that curvature could also represent indecision between the response alternatives in mouse-tracking paradigms.

In addition to curvature, time-continuous analyses of mouse movements make it possible to dissect how different factors affect mouse movement at different points in time (Dshemuchadse et al., 2013; Scherbaum et al., 2010). Here the angle of a mouse movement at different time points can be used to assess general or transient response tendencies. In addition to angle and curvature, which reflect the movement direction, the speed of the mouse cursor is recorded. The interpretation of speed depends on the experimental manipulation. For example, speed has been used in previous studies to assess uncertainty (Hehman et al., 2015), cognitive load (Rheem et al., 2018), and the presence of task-unrelated thoughts (Da Dias Silva & Postma, 2020).

Mouse tracking thus allows the examination of two elementary classes of measures. First, measures related to the direction of movement (curvature and movement angle) and second, the speed of this movement. Due to the different measures for direction and speed of movement, which indicate underlying processes, mouse tracking is beneficial for current questions in PM research.

Concerning the dissection between spontaneous retrieval processes, a recent study investigating the effects of task-unrelated thoughts on mouse movement direction and speed provides interesting findings (Da Dias Silva & Postma, 2020). In this study, task-unrelated thoughts reduced movement speed but did not affect the magnitude of movement deviation. In our view, similarly, the memory search, as postulated by the discrepancy plus search process, implies thoughts unrelated to the ongoing task. In contrast, the reflexive-associative process is not assumed to be accompanied by task-unrelated thoughts. Instead, the PM cue is assumed to reflexively trigger the PM response, presumably leading to simultaneous activation of the ongoing-task response and the PM response, which would elicit a response conflict. In mouse-tracking studies, such a response conflict is reflected in a change of movement direction, which is reflected in the direction measures curvature and angle (Calcagnì et al., 2017; Kieslich & Hilbig, 2014; Scherbaum et al., 2010; Spivey et al., 2005; Spivey & Dale, 2006).

According to capacity-sharing theories, the presence of a PM task imposes a type of cognitive load that leads to less efficient processing of ongoing task stimuli, as is reflected in a reduced drift rate in evidence accumulation models (e.g., Anderson et al., 2018). In mouse tracking, these processes should show up in reduced speed, as evidenced by a recent study investigating the influence of cognitive load on movement deflection and speed (Rheem et al., 2018). In this study, a high cognitive load in a secondary task resulted in slower movement speed in a primary task. Interestingly, curvature was also reduced in the high load condition. In other words, sharing cognitive capacity between two tasks resulted in slower but more direct (in terms of movement direction) response behavior.

How would a higher response threshold, as postulated by the delay theory, affect mouse movements? A delayed response means that a decision for a response is made relatively late in a trial. In other words, there is more prolonged indecision between the response alternatives. In mouse tracking paradigms, indecision between the response alternatives typically means keeping the mouse movement in the center of the screen and not targeting any response box (Erb, 2018; Fischer & Hartmann, 2014). This prolonged staying in the middle during the movement is reflected in the direction measures (i.e., curvature and angle).

To the best of our knowledge, so far only two studies have been published that combined mouse tracking with a PM task (Abney et al., 2015; Hicks et al., 2019). Abney et al. (2015) focused on whether and under what conditions a PM task can be spontaneously retrieved or to require additional cognitive processes to support intention retrieval. By comparing speed profiles of mouse movements in PM tasks with varying cue focality, Abney et al. (2015) found evidence for capacity-sharing processes in PM tasks with non-focal PM cues that are associated with higher ongoing-task costs than focal PM cues (Einstein et al., 2005). Here, capacity sharing manifested itself in a delayed onset of the mouse-movement speed in the condition with non-focal PM cues.

Like Abney et al. (2015), Hicks et al. (2019) varied cue focality in a mouse-tracking setup. In addition to speed, they investigated the initiation time of mouse movement (i.e., the time it takes a participant to start the mouse movement) and how directly a movement associated with the PM response was performed in both conditions. Due to the arrangement of the response boxes for the ongoing task and the PM task in the experimental setup of their study, Hicks and colleagues were able to investigate not only the speed of mouse movement but also its curvature to infer cognitive processes. In the non-focal condition, the mouse movement started later and had a greater curvature than in the focal condition. Similar to Abney et al. (2015), Hicks et al. (2019) interpreted their results as evidence for increased capacity-sharing in non-focal conditions.

Taken together, the speed and direction of mouse movement have previously been used to infer cognitive processes in general and to a lesser extent in PM tasks. As we outline in the following section, our paradigm has the advantage of allowing for the time-continuous analysis of the speed of mouse movement and its direction for each individual task and their interaction. Another advantage of our paradigm is a dynamic start condition (Schoemann et al., 2019) and a dynamic stimulus presentation that allow participants to move the mouse across the screen with greater freedom than previous mouse-tracking studies. Thereby, our paradigm is suited to dissect the sub-processes of spontaneous retrieval besides giving detailed descriptions of the processes underlying costs to the ongoing task.

#### Paradigm

To investigate the processes underlying aftereffects of completed intentions and costs to the ongoing task, we implemented a mouse-tracking version of the repeated PM cue paradigm that contained several experimental cycles, each consisting of an active phase with an event-based PM task and a subsequent finished phase in which PM cues from the recently finished PM task were presented as PM_REPEATED_ cues (Walser et al., 2012; Walser et al., 2017). In all cycles, the participants’ ongoing task was to perform digit categorizations by moving the mouse cursor in one of two response boxes in the upper left corner of the screen. The PM task required participants to move the mouse cursor in the upper right corner of the screen instead of performing the digit categorization in response to a pre-specified PM cue (geometric shape surrounding the digit). Deviating from the original repeated PM cue paradigm, finished phases were assigned to one of two conditions. In the *PM-task-repetition* condition, subjects performed a novel but similar PM task (PM cue: different geometric shape) during the finished phase. In the *ongoing-task only* condition, subjects only performed the ongoing task during the finished phase. Concerning this design, it can be argued that the alternation of phases could impact the effects underlying spontaneous retrieval or costs to the ongoing task. However, previous studies have successfully used similar paradigms to investigate PM processes (e.g., Walser et al., 2012; Walser et al., 2017).

This design enabled us to assess aftereffects of completed intentions and the effects of engaging in a novel PM task after intention completion on aftereffects. Simultaneously, it made it possible to assess ongoing-task costs that can occur during the performance of PM tasks by comparing ongoing-task performance between finished phases with and without an event-based PM task. Note that previous research recommended using PM paradigms with highly salient focal PM cues to investigate spontaneous retrieval of active intentions (e.g., McDaniel et al., 2015) and to exacerbate aftereffects of completed intentions (e.g., Bugg & Streeper, 2019; Möschl et al., 2020), and to use non-focal PM cues to exacerbate ongoing-task costs (e.g., Scullin et al., 2010). While our use of salient non-focal PM cues in the present study deviates from these recommendations, Walser et al. (2017) showed that salient non-focal cues reliably produce RT aftereffects as well as ongoing-task costs in a repeated PM cue paradigm. Therefore, we consider our choice of PM cues a suitable compromise that enabled us to assess both spontaneous retrieval of completed intentions and ongoing-task costs within the same paradigm. Furthermore, this choice also allows us to study the interaction of both processes.

*Aftereffects of completed intentions* – *hypotheses*. In line with previous research (Walser et al., 2012; Walser et al., 2017), we expected aftereffects in terms of overall slower ongoing-task responses in PM_REPEATED_ trials compared to oddball trials during finished phases. Furthermore, to investigate the role of spontaneous retrieval as a cause of aftereffects of completed intentions, the ongoing-task-only condition was of particular interest since it did not require performing a PM task in addition to the ongoing task.

We hypothesized that if spontaneous retrieval of completed intentions was primarily caused by bottom-up reflexive-associative processes, PM_REPEATED_ trials should lead to a spontaneous reactivation of the PM response linked to the PM_REPEATED_ cue. More specifically, we argue that this would induce a response conflict and interfere with the ongoing-task response. In line with previous findings of low commission-error rates in the repeated PM-cue paradigm (Walser et al., 2012; Walser et al., 2014; Walser et al., 2017), we expect that in most PM_REPEATED_ trials, the intention would not be executed in full but that the corresponding movement would be interrupted beforehand in order to give an ongoing-task response. Furthermore, in mouse-tracking data, this response conflict between PM response and ongoing-task response should primarily result in greater curvature of mouse movements toward the PM response box in PM_REPEATED_ trials than in oddball trials. In other words, the reflexively retrieved and subsequently interrupted PM response should lead to a deviation in the otherwise straight ongoing-task response.

Alternatively, if spontaneous retrieval of completed intentions was primarily caused by a discrepancy-plus-search process, PM_REPEATED_ trials should trigger an experience of discrepancy and a subsequent search in memory for the cause of this discrepancy. In contrast to reflexive-associative retrieval, we do not expect a discrepancy-plus-search process to induce a response conflict. Instead, we argue that the discrepancy induced by the PM_REPEATED_ cue and the subsequent search for the cause of the discrepancy does not necessarily end in retrieving the intended action, but instead, the memory search may result in the realization that the intention has already been completed, for example, because it is tagged with a stop-tag as no longer to be executed or because the action has been disassociated and is now no longer linked to the PM cue (Bugg & Scullin, 2013; Streeper & Bugg, 2021).

In our view, the experienced discrepancy and the memory search result in thoughts irrelevant to the ongoing task and should therefore primarily result in a decreased speed of mouse movements toward ongoing-task response boxes in PM_REPEATED_ trials compared to oddball trials. Furthermore, in contrast to a reflexive-associative process, a discrepancy-plus-search process should not induce a response conflict or alter response tendencies because the intended action is not reflexively retrieved. Thus, there should be no differences in the curvature and angle of mouse movements between PM_REPEATED_ and oddball trials.

Additionally, we expect that engaging in a novel PM task during the finished phase should exacerbate aftereffects of completed intentions and make effects of the underlying sub-processes of spontaneous retrieval of completed intentions more pronounced (Walser et al., 2017). Spontaneous intention retrieval due to reflexive-associative processes should result in a more substantial difference in curvature between PM_REPEATED_ and oddball trials in the PM-task-repetition condition than in the ongoing-task-only condition. Likewise, spontaneous intention retrieval due to discrepancy-plus-search processes should lead to more substantial differences in speed between PM_REPEATED_ and oddball trials in the PM-task-repetition condition than in the ongoing-task-only condition.

*Costs to the ongoing task* – *hypotheses.* In line with previous research, regarding our discrete performance measures, we expected ongoing-task costs in terms of slower ongoing-task responses in standard trials during finished phases in the PM-task-repetition condition compared to the ongoing-task-only condition (Einstein & McDaniel, 2010; McDaniel & Einstein, 2000; Smith, 2003; Smith, 2010; Smith et al., 2007; Smith & Bayen, 2004). Regarding our continuous measures, we based our predictions on both theoretical considerations and findings from recent modeling studies (e.g., Anderson et al., 2018; Heathcote et al., 2015) that aimed to shed light on the source of response slowing during the pursuit of PM intentions.

On the one hand, capacity-sharing theories attribute ongoing-task costs to the additional load imposed by a PM task and slower evidence-accumulation rates (Boywitt & Rummel, 2012; Horn et al., 2011; Smith, 2003; Smith et al., 2007), which we assume to be reflected in the speed of mouse movements. At the same time, findings from hand-movement tracking (Rheem et al., 2018) showed that imposing additional load on a primary task by adding a secondary task resulted in overall slower movement speed but had no effect on movement curvature or angle. Therefore, we expect that if ongoing-task costs were due to sharing of limited capacity between tasks, pursuing a PM task should affect movement speed but not the movement curvature or angle. More specifically, we argue that movement speed should be slower in the PM task repetition condition than in the ongoing-task-only condition, while there should be no differences in movement curvature or angle between conditions.

On the other hand, delay theory attributes ongoing-task costs to a higher response threshold for the ongoing task without changes to the rate of evidence accumulation for the ongoing task (Heathcote et al., 2015; Loft & Remington, 2013; Strickland et al., 2017). A higher response threshold is identical with more extended indecision between response alternatives, which would show up in a mouse movement that does not target a specific response box but follows the middle of the screen (Erb, 2018; Fischer & Hartmann, 2014). Therefore, we expect that if ongoing-task costs resulted from a strategic delay of ongoing-task responses via heightened response thresholds, pursuing a PM task should result in a mouse movement that remains more prolonged in the middle of the screen than in the ongoing-task-only condition. Hence, movement curvature should be more pronounced, and movement angles should be smaller in the PM-task-repetition than in the ongoing-task-only condition.

Note that, in our paradigm, it cannot be ruled out that a strategic delay of responses affects not only movement direction but also movement speed. Thus, for example, one could argue that indecision between responses results in a reduced speed (additionally to a movement that remains in the middle between response boxes). Consequently, we cannot distinguish between delay and capacity-sharing theories when considering speed only. Both theories are consistent with a movement slowing in the PM-task-repetition condition. However, the delay theory also predicts that this slowing is associated with a higher response threshold in the PM-task-repetition condition, which would show up in a mouse movement (evident in curvature and movement angle) that does not target a specific response box but stays longer in the middle of the screen. In contrast, capacity-sharing theories predict no such change in the movement direction.

## Methods

### Participants

Forty-two participants (28 female; *M*_age_ = 23 years, *SD*_age_ = 3.4 years) were recruited through the department’s database system, based on ORSEE (Greiner, 2004). All participants had normal or corrected-to-normal vision and a right-handed mouse movement in everyday life. Participants declared prior to the experiment that they had no acute psychiatric disorders and were not currently exposed to any extraordinary stress in their lives. Due to a lack of previous studies that used mouse-tracking to assess aftereffects of completed intentions and ongoing-task costs of performing PM tasks, we based our sample size calculation on discrete effects. Since we modeled our experiment after Experiment 3 of the Walser et al. (2017) study and aimed to replicate the discrete effects from this experiment, we aimed for a sample size about 2.5 times larger than in their study (*N* = 16) (Simonsohn, 2015). In order to meet balancing constraints of our study, we aimed to collect a sample of *N* = 42 subjects, which enabled us to detect effects of at least *d* = 0.44 at 80% power in a two-tailed repeated-measures *t*-test at alpha = .05 (G-Power, Version 3.1.9.6; Faul et al., 2007).

### Apparatus and stimuli

The experiment was running on a personal computer with the Windows 7 operating system. We used the Psychophysics Toolbox (Version 3.12) (Brainard, 1997; Kleiner et al., 2007; Pelli, 1997) in Matlab 2010 (The Mathworks Inc., Natick, MA, USA). Stimuli were presented on a 19-in. (3:4 format) screen with a resolution of 1,280 × 1,024 pixels and a refresh rate of 75 Hz. The screen was positioned about 60 cm in front of participants. Responses were given with a corded computer mouse (Logitech Laser Mouse USB). The pointer speed of mouse motion was set to 4, and the enhanced pointer precision was switched off in the control panel of the Windows operating system. All stimuli and response boxes were presented in black on a grey background. Ongoing-task stimuli were digits 2–9 presented in Arial font with a font size of 75 pt. Symbols for PM stimuli and oddballs were primarily similar to those used in Experiment 3 of the study by Walser et al. (2017).^3^ An illustration of all symbols can be found in the appendix.

### Procedure and design

The duration of an experimental session was about 1 h. Participants received 8 € for their participation. The study was performed in accordance with the guidelines of the Declaration of Helsinki and the German Psychological Society. Ethical approval was not required since the study did not involve any risk or discomfort for the participants. All participants were informed about the study's purpose and procedure and gave written informed consent prior to the experiment. All data were analyzed anonymously.

**Fig. 1:**
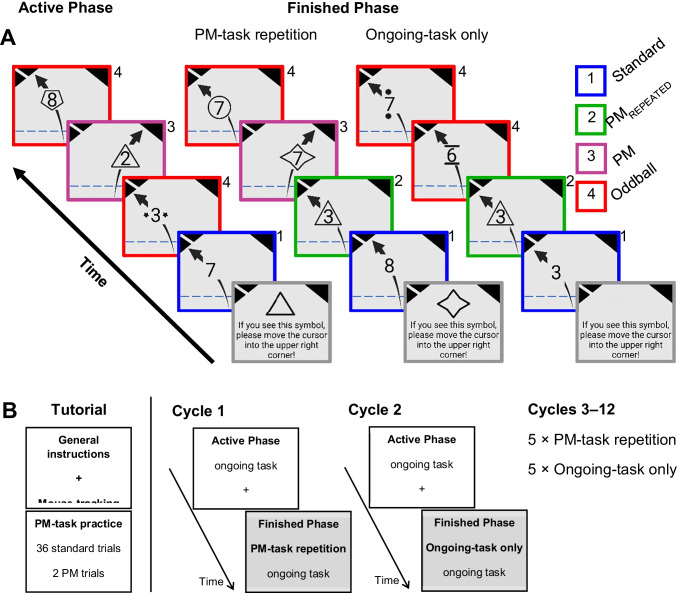
Procedure. **a** Example trials of the active phase with a prospective memory (PM) task and the finished phases with or without a PM task are shown. In all trials except for PM trials, participants had to categorize digits according to parity by moving the mouse cursor from the lower edge of the screen into the corresponding response boxes at the upper-left of the screen (i.e., the lower response box for odd numbers and the upper one for even numbers). In the active phase, participants had to respond to specific symbols (e.g., triangle), which served as PM cues, by moving the mouse cursor into the response box at the upper-right of the screen. In finished phases, participants either performed a PM-task-repetition condition or an ongoing-task-only condition. In the PM-task-repetition condition, they performed another PM task in which they had to give a PM response to a different symbol than the PM cue from the active phase. In the ongoing-task-only condition, they had to perform only the ongoing task in all trials. Aftereffects of completed intentions were assessed during finished phases by comparing ongoing-task-performance and commission error rates in PM_REPEATED_ trials and oddball trials. Standard trials were trials without an additional symbol surrounding the target digit and required only an ongoing-task response. Note that the framing of trial types was not present in the experiment but serves exclusively to illustrate different trial types in this figure. **b** Schematic representation of the procedure. The experiment started with Instructions and practice of the mouse-tracking procedure, followed by a brief practice of the PM task. After that, participants performed 12 experimental cycles that each consisted of an active phase and a finished phase. Participants alternated between cycles with a finished phase in the PM-task-repetition condition and cycles with a finished phase in the ongoing-task-only condition. One half of the participants started the experiment with a cycle in the PM-task-repetition condition, while the other half started with a cycle in the ongoing-task-only condition

In the experiment, a number categorization task served as an ongoing task. In this task, participants had to indicate whether the presented digit (2–9) was even or odd by moving the mouse cursor into one of two triangular response boxes in the upper-left corner of the screen (Fig. 1). Participants had to move the cursor in the lower response box as a response to an even number. In trials where an odd number appeared, they had to move the cursor in the upper response box. The PM task was to move the mouse cursor in a triangular response box in the screen's upper-right corner when a specific symbol appeared (i.e., PM cues; e.g., triangle). PM cues were presented together with the stimuli of the ongoing task.

Each experimental session started with a tutorial in which participants first practiced the course of a single trial in small stages. For this purpose, blue arrows were presented on the screen in addition to stimuli and response boxes to show where subjects should maneuver the mouse cursor next. After that, participants received the instruction for the ongoing task and practiced ten standard trials (i.e., only the ongoing task stimuli were present). Subsequently, participants received the PM task instruction and practiced it during 38 trials (36 Standard, two PM). After the tutorial, the participants completed twelve cycles consisting of an active phase followed by a finished phase (Möschl et al., 2020) (Fig. 1). After each phase, participants could take a self-paced break (at least 5,000 ms). The active phase served to assess PM performance. The finished phases served to assess aftereffects and/or costs to the ongoing task. Six finished phases served as a PM-task-repetition condition, and six finished phases served as an ongoing-task-only condition. While in the PM-task-repetition condition, participants had to perform a new PM task, in the ongoing-task-only condition, no new PM task had to be performed.

At the beginning of each cycle, the participants received task instructions. In the active phase and the PM-task-repetition condition of the finished phase, the instruction was to move the mouse cursor in the screen's upper-right corner upon the occurrence of a specific symbol (i.e., the PM cue). These symbols were shown on the instruction screen. Active phases included 40 standard trials, four PM trials, and four oddball trials (i.e., digits with an additional symbol that never served as PM cues during the entire experiment). In the PM-task-repetition condition, the finished phase contained 40 standard trials, four PM_REPEATED_, four oddballs, and four new PM trials. In the ongoing-task-only condition, participants were instructed not to respond to any symbol shown. This phase included 40 standard trials, four PM_REPEATED_ trials, and eight oddball trials. Note that oddball trials in the ongoing-task-only condition were two different oddball cues that were presented four times each). Here we included two oddball cues to ensure that the same number of deviant stimuli appeared as in the PM-task-repetition condition. In standard trials, each digit was randomly selected for each trial. For each participant, symbols were randomly assigned to trial types and blocks. Trial types in each block were presented in randomized order with the following restrictions. First, each block started with two standard trials. After that, each trial in which a symbol was present was followed by two standard trials.

We used a dynamic start procedure of tracking mouse movements to support the mapping of continuous cognitive processes in mouse trajectories by preventing participants from finishing cognitive processes of interest before moving the mouse (Grage et al., 2019; Scherbaum & Kieslich, 2017). Each trial consisted of three stages. In the *start stage*, participants started each trial by moving the mouse cursor (crosshair) to the screen's lower edge. After that, the cursor was set to the middle of the lower screen border (coordinates 640/10 pixels). After participants moved the mouse upwards 160 pixels along the Y-axes, the *response stage* started, and the mouse cursor was transformed into the target stimulus of the respective trial. At the same time, three response boxes appeared in the upper corners of the screen. Each trial was finished by moving the mouse cursor in one of the three response boxes, thereby categorizing the stimulus correctly. Hereafter, the *reset stage* started, and the mouse cursor was reconverted into a crosshair, and participants had to move it back to the lower edge of the screen to start the subsequent trial. The maximum duration of a full trial was 4,500 ms (1,500 ms per stage). If the movement was not finished after the corresponding limits, a feedback tone (450 Hz) was delivered for 150 ms through headphones, and an error message was presented in red font and German language. In case of exceeding the maximum duration of the start stage, the error message was *"Zu langsam zum Start gegangen"* (Moved too slowly to the start). After the response stage, the error message read *"Zu spät gestartet"* (Start was too late), and after the reset stage, the error message was *"Zu langsame Entscheidung"* (The decision was too slow). If participants moved the mouse to an incorrect response box, the trial ended immediately, and a different feedback tone (720 Hz) was presented for 150 ms without additional visual feedback. During the whole experiment, X/Y-coordinates of the mouse cursor were tracked with a sample rate of 100 Hz.

### Data preprocessing and analysis

To investigate the processes underlying spontaneous retrieval of completed intentions, we compared mouse-movement data in PM_REPEATED_ trials to oddball trials in finished phases. To investigate costs to the ongoing task, we compared mouse-movement data in standard trials between finished phases of the PM-task-repetition condition and the ongoing-task-only condition. Only the data from the response stage were included in further analyses. For the analysis of ongoing-task costs, we followed the procedure of previous studies and excluded one standard trial that followed PM, oddball, and PM_REPEATED_ trials (Meier & Rey-Mermet, 2012; Walser et al., 2014), which were on average 19.29% of all trials of a participant. For the analyses of RT data and continuous effects, we excluded erroneous responses (3.03%) in standard trials and commission errors (i.e., PM responses in PM_REPEATED_ trials, 1.49%). Additionally, for each participant, we removed all trials in which RTs for a correct response exceeded an outlier criterion of three standard deviations above or below the mean RT of a participant per trial type in each condition. On average, 1.74% of trials of each participant were identified as outliers. Mouse-movement data after stimulus onset was normalized into 100 equal time steps (Dshemuchadse et al., 2013; Scherbaum et al., 2010; Spivey et al., 2005). From the X-coordinate at each time step, 640 pixels (half of the total number of horizontal pixels) were subtracted (X-coordinate of the start point = 0). Consequently, negative X-coordinates indicate a movement in the direction of the ongoing-task response box, and positive X-coordinates indicate a movement to the PM response box. We calculated the area under the curve (AUC) of each trial as a measure for curvature. We then transformed AUC data into *z* scores separately for each participant over all trials of the compared conditions (Dshemuchadse et al., 2013; Scherbaum et al., 2010; Spivey et al., 2005). Data preprocessing and analyses of continuous measures were performed in Matlab 2020 (The Mathworks Inc., Natick, MA, USA). ANOVAs, *t*-tests, and correlation analyses were performed in JASP (Version 0.13.1). Figure 5 was created with gramm (Morel, 2018).

## Results

First, we overview discrete measures of PM performance in the active phase. Note, the results of the active phase are presented for descriptive reasons only and are not included in further analyses. Second, we present our analyses of discrete performance measures (i.e., mean RTs and error rates) of aftereffects and costs to the ongoing task. The analyses of discrete effects served as a first step to investigate whether we could replicate previous findings on aftereffects of completed intentions, the effects of a new PM task on aftereffects, and costs to the ongoing task in our mouse-tracking paradigm. Third, we present our analyses of the continuous measures of mouse tracking that we used to assess sub-processes underlying the effects we observed in our discrete performance measures. In both the discrete and continuous analyses, the results on aftereffects are presented first, followed by the results on costs to the ongoing task.

### Discrete effects

**Fig. 2:**
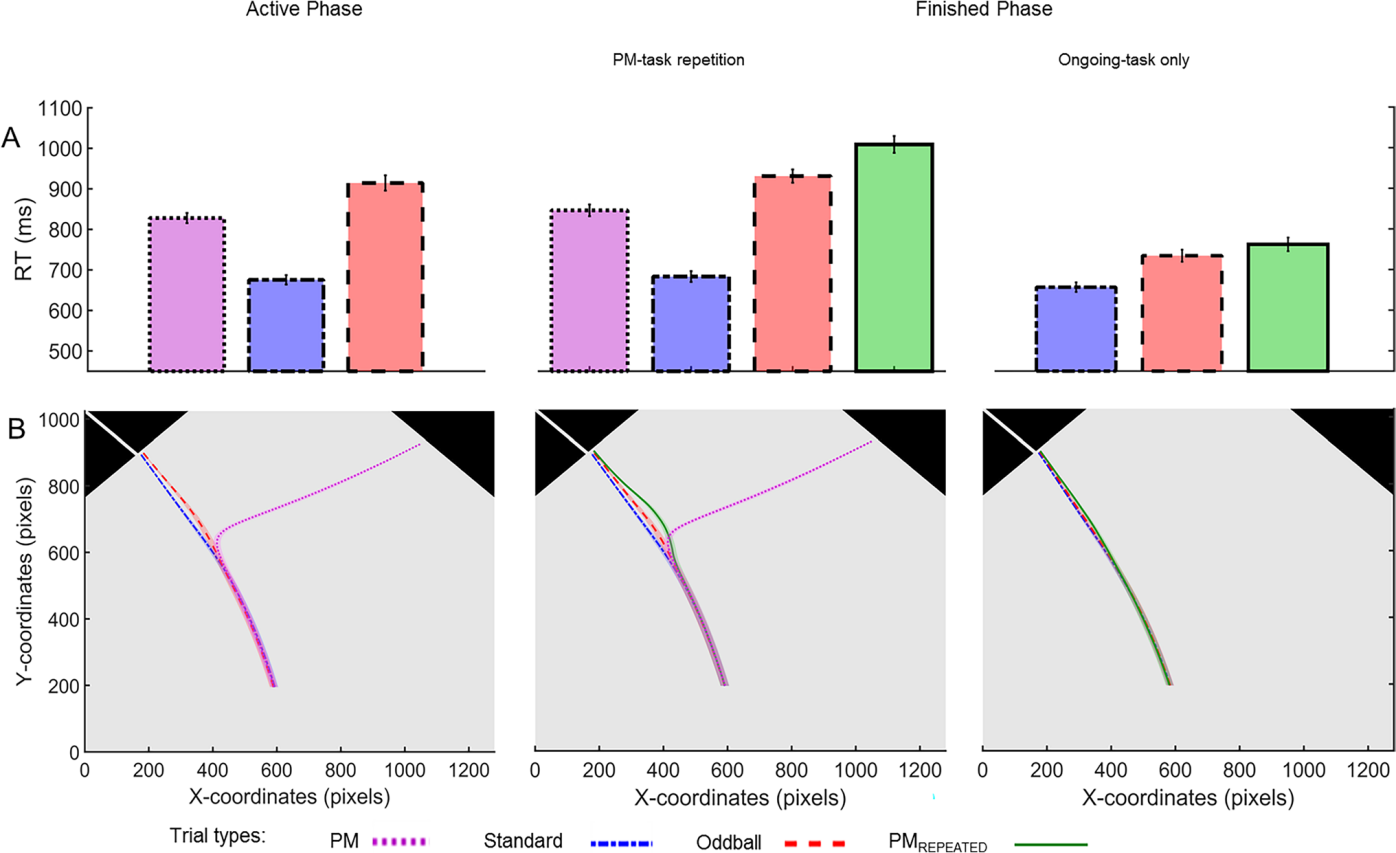
**a** Mean response times (RT) and **b** mouse trajectories (x and y coordinates) as a function of trial type in the active phase and as a function of finished phase condition (PM-task-repetition and ongoing-task only) and trial type in the finished phase. In panel (**a**) error bars represent standard errors. In panel (**b**), confidence areas indicate the standard error in the respective time step. PM = prospective memory

The results of the discrete-effects analyses are shown in Fig. 2a.

#### Active phase performance

Mean RTs were 828 ms (*SD* = 81 ms) in PM trials, 915 ms (*SD* = 122) in oddball trials, and 676 ms (*SD* = 75 ms) in standard trials. Mean error rates were 5.56% (*SD* = 5.63%) in PM trials, 9.87% (*SD* = 5.54%) in oddball trials and 3.03% (*SD* = 2.64%) in standard trials.

#### Aftereffects of completed intentions

We conducted a 2 (trial type: PM_REPEATED_, oddball) × 2 (finished-phase condition: PM-task repetition, ongoing-task only) repeated-measures ANOVA to assess aftereffects of completed intentions in RTs and commission errors.

*RTs.* Participants responded slower in PM_REPEATED_ (*M* = 886 ms, *SD* = 174 ms) as compared to oddball trials (*M* = 833 ms, *SD* = 141 ms), indicating aftereffects of completed intentions, *F*(1, 41) = 35.51, *p* < .001, η_*p*_^2^ = .46. Response times were slower in the PM-task-repetition condition (*M* = 971 ms, *SD* = 127 ms) than in the ongoing-task-only condition (*M* = 748 ms, *SD* = 103 ms), *F*(1, 41) = 627.26, *p* < .001, η^2^= 0.94. Further, aftereffects were increased in the PM-task-repetition condition (*M* = 78 ms; *t*(41) = 5.76 , *p* < .001, *d* = 0.89, 95% CI [0.53, 1.24]) as compared to the ongoing-task-only condition (*M* = 28 ms; *t*(41) = 3.32, *p* = .002, *d* = 0.51, 95% CI [0.19, 0.83])), as revealed by a Test-block condition × Trial type interaction, *F*(1, 41) = 13.05, *p* < .001, η_*p*_^2^ = 0.24.

*Commission errors.* A commission error was counted as soon as the mouse cursor was moved into the PM response box in a PM_REPEATED_ trial. In this case, the trial ended immediately. We calculated the percentage of commission errors for each subject based on the commission errors made and the total number of PM_REPEATED_ trials. Participants made more commission errors in PM_REPEATED_ trials (*M* = 1.49%, *SD* = 2.79%) than in oddball trials (*M* = 0.55%, *SD* = 1.92%), indicating aftereffects of completed intentions, *F*(1, 41) = 11.59, *p* = .001, η_*p*_^2^ = 0.22. Overall, commission errors were more frequent in the PM-task-repetition condition (*M* = 1.84%, *SD* = 3.13%), than in the ongoing-task-only condition (*M* = 0.20%, *SD* = 0.89%), *F*(1, 41) = 16.34, *p* < .001, η_*p*_^2^ = 0.29, but commission-error aftereffects did not differ significantly between conditions, *F*(1, 41) = 3.27, *p* = .078, η_*p*_^2^ = 0.07.

#### Costs to the ongoing task

To investigate costs to the ongoing task, we performed paired *t*-tests to compare RTs and error rates on standard trials during finished phases between the PM-task-repetition condition and the ongoing-task-only condition. Participants responded slower in the PM-task-repetition condition (*M* = 684 ms, *SD* = 87 ms) than in the ongoing-task-only condition (*M* = 657 ms, *SD* = 77 ms), *t*(41) = 6.46, *p* < .001, *d* =1.00, 95% CI [0.62, 1.36]. This finding indicates that the additional PM task in the PM-task-repetition condition entailed costs to the ongoing task (Smith, 2003). Error rates were lower in the PM-task-repetition condition (*M* = 2.68%, *SD* = 2.25%) than in the ongoing-task-only condition (*M* = 3.37%, *SD* = 2.68%), as revealed by the paired t-test, *t*(41) = -2.84, *p* = .007, *d* = -0.44, 95% CI [-0.75, -0.12].

### Continuous effects

**Fig. 3:**
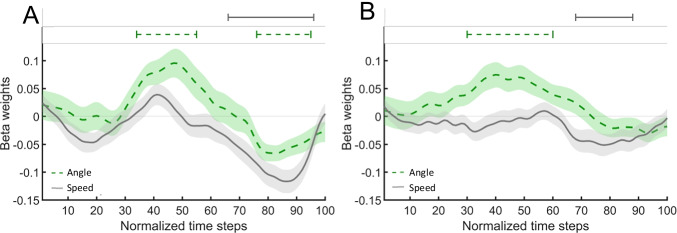
Results of continuous regression analyses on mouse movement in PM_REPEATED_ vs. oddball trials in the (**a**) PM-task-repetition condition and (**b**) ongoing-task-only condition of the finished phase. Dashed lines indicate the angle of the mouse movement. Grey, solid lines indicate the speed of mouse movement. For the movement angle, positive beta weights indicate a stronger orientation of movements toward the PM-response box in PM_REPEATED_ than in oddball trials; negative values indicate a stronger movement orientation toward the ongoing-task response boxes in PM_REPEATED_ than in oddball trials. For movement speed, positive beta weights indicate faster movements in PM_REPEATED_ than in oddball trials. Negative beta weights indicate slower movements in PM_REPEATED_ than in oddball trials. Lines above the graphs indicate segments of beta weights that were significantly different from zero (consecutive *t*-test; only segments with a minimum of ten consecutive significant time steps are shown). Confidence areas indicate standard errors of beta weights

Mouse trajectories are shown in Fig. 2b. Results of continuous regression analyses on mouse-movement data are depicted in Fig. 3 (aftereffects of completed intentions) and Fig. 6 (costs to the ongoing task). We performed time-continuous linear regression analyses on movement angles and speed to investigate the mouse movements' temporal dynamics. We calculated movement angles to measure the direction of mouse movements in the segment between two consecutive time steps. For this, we first calculated the vector between the coordinates of two consecutive time steps and calculated the angle between this vector and the Y-axis. After that, we standardized (-1 to 1) angles for each participant (Scherbaum & Kieslich, 2017). Thus, a movement in the direction of the ongoing-task responses is represented by negative movement angles, and positive movement angles represent a movement in the direction of the PM response box. For the statistical comparison of movement angles, we performed multiple regression analyses on the angles of all consecutive time steps (Dshemuchadse et al., 2013; Notebaert & Verguts, 2007; Scherbaum et al., 2010; Scherbaum & Kieslich, 2017). The speed of the mouse movement was calculated as the magnitude of the difference vector between two consecutive time steps. For the statistical analyses of speed data, we performed the same procedure as on movement angles.

### Aftereffects of completed intentions

**Fig. 4:**
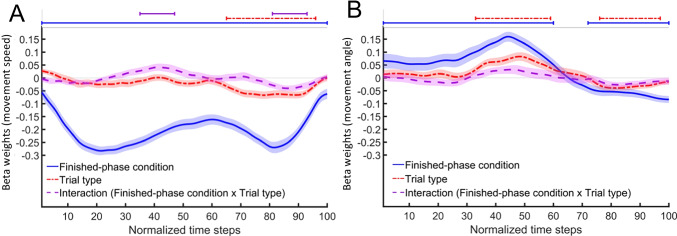
Results of continuous regression analyses on mouse movement in PM_REPEATED_ vs. oddball trials (dash-dotted line) and in PM-task-repetition condition vs. ongoing-task only condition (solid line) with the dashed line showing the interaction of condition and trial type. Continuous regression analyses were performed on speed (**a**) and angle (**b**) of mouse movement. Lines above the graphs indicate segments of beta weights that differ significantly from zero (t-test, a minimum of ten consecutive significant time steps). Confidence areas indicate standard errors of beta weights. The positive/negative characteristics describe the direction of effects. Positive beta weights signify larger values in PM_REPEATED_ than in oddball trials (dash-dotted line), respectively, in the PM-task-repetition condition than in the ongoing-task-only condition (solid line). Negative beta weights signify smaller values in PM_REPEATED_ than in oddball trials (dash-dotted line), respectively, in the PM-task-repetition condition than in the ongoing-task-only condition (solid line). The positive interaction (dashed line) in the analysis of speed in the second third of a trial suggests inverse aftereffects in this segment. However, note, this interaction does not result in a significant main effect of the factor trial type in this segment. The negative interaction in the last third indicates increased aftereffects in the corresponding segment

Since we are interested in the processes underlying spontaneous retrievals of completed intentions in the absence of a strategic delay or capacity-sharing processes, we first report the results of the analyses of the angle and speed of mouse movements in the ongoing task only condition (Fig. 3b). Figure 3a shows similar analyses in the PM task repetition condition. To statistically investigate how the presence of a new PM task affected retrieval processes, we then report the results of the interaction of finished-phase condition and trial type (Fig. 4).

To elucidate the role of sub-processes of spontaneous retrieval of completed intentions, we first compared movement curvatures in PM_REPEATED_ trials and oddball trials of the ongoing-task-only condition in a paired *t*-test. We found greater curvature in PM_REPEATED_ trials (*M*_*z-score*_ = -0.07, *SD* = 0.15) than in oddball trials (*M*_*z-score*_ = -0.13, *SD* = 0.12), *t*(41) = 2.04, *p* = .048, *d* = 0.32, 95% CI [0.003, 0.62]. Subsequently, we performed time-continuous regression analyses on the angle and speed of mouse movements with the trial type (PM_REPEATED_, oddball) as the predictor variable (Fig. 3b). Time segments with consecutive significant beta weights are shown in Table 1. This analysis revealed that only during the second third of a trial (time steps 30–60) mouse movements in PM_REPEATED_ trials were angled more in the direction of the PM response box than in oddball trials. Our analysis of movement speed showed that this movement deflection was followed by a more pronounced response slowing in PM_REPEATED_ trials than oddball trials in the last quarter of a trial (time steps 68–88).

**Table 1:** Consecutive time segments of significant beta weights in time continuous regression of the angle and the speed of mouse movement in PM_REPEATED_ trials as compared to oddball trials

	PM-task-repetition condition		Ongoing-task-only condition	
	Movement angle	Movement speed	Movement angle	Movement speed
Consecutive significant time steps (*p* < .05)	[34, 55], positive [76, 95], negative	[66, 96], negative	[30, 60], positive	[68, 88], negative

*Note.* Numbers in brackets correspond to the start and endpoint of consecutive time-series segments in which mouse-movement characteristics differed significantly between PM_REPEATED_ and oddball trials ([start segment, end segment]). The positive/negative characteristics describe the direction of these differences. Positive differences signify a larger value in PM_REPEATED_ than in oddball trials. Negative differences signify smaller values in PM_REPEATED_ than in oddball trials. PM = prospective memory

In order to assess the effects of performing another PM task after intention completion on intention deactivation, we compared aftereffects in mouse-movement data between finished-phase conditions. First, we conducted a 2 (trial type: PM_REPEATED_, oddball) × 2 (finished-phase condition: PM-task repetition, ongoing-task only) repeated measures ANOVA on *z*-transformed AUC data to compare aftereffects on curvature. We found a greater curvature in the PM-task-repetition condition (*M*_*z-score*_ = 0.17, *SD* = 0.24) than in the ongoing-task-only condition (*M*_*z-score*_ = -0.10, *SD* = 0.14), *F*(1, 41) = 46.49, *p* < .001, η_*p*_^2^ = 0.53. In PM_REPEATED_ trials curvature was greater (*M*_*z-score*_ = 0.09, *SD* = 0.26) than in oddball trials (*M*_*z-score*_ = -0.02, *SD* = 0.21), *F*(1, 41) = 16.48, *p* < .001, η_*p*_^2^ = 0.29, indicating that participants moved the mouse more into the direction of the PM-response box in PM_REPEATED_ trials than in oddball trials. In contrast to RT analyses, finished-phase condition and trial type did not interact, *F*(1,41) = 2.92, *p* = .095, η_*p*_^2^ = 0.06.

Subsequently, we conducted time-continuous regression analyses on the angle and speed of mouse movement data with the finished-phase condition (PM-task repetition, ongoing-task only) and trial type (PM_REPEATED_, oddball) as predictor variables (Fig. 4). Table 2 shows time segments with significant beta weights. Our analysis of movement angles revealed a more substantial movement deflection toward the PM-task response box in the PM-task-repetition condition than in the ongoing-task-only condition during the first two thirds of a trial (time steps 1–61), followed by a more substantial compensation toward the ongoing-task response boxes over the last quarter of a trial (time-steps 72–100) in the PM-task-repetition condition. Mouse movements in PM_REPEATED_ compared to oddball trials showed a deflection toward the PM-task response box during the second third of a trial (time steps 33–59), followed by a deflection toward the ongoing-task response boxes during the last quarter of a trial (time steps 76–97). We did not find any significant Finished-phase condition × Trial type interaction.

**Table 2:** Consecutive time segments of significant beta weights in time continuous regression of the angle and the speed of mouse movement in time-continuous regression analyses with Finished-phase condition and Trial type as predictor variables

	Predictor variables	Movement angle	Movement speed
Consecutive significant time steps (p < .05)	PM-task-repetition condition vs. ongoing-task-only condition	[1, 61], positive [72, 100], negative	[1, 100], negative
	PM-repeated trials vs. oddball trials	[33, 59], positive [76, 97], negative	[65, 96], negative
	Finished-phase condition × Trial type interaction		[35, 47], positive [81, 93], negative

*Note.* Numbers in brackets correspond to the start and endpoint of consecutive time-series segments of beta weights that differ significantly from zero ([start segment, end segment]) in time continuous regression analyses on the angle and speed of mouse movement with finished-phase condition and trial type as predictor variables. Positive beta weights signify larger value in PM_REPEATED_ than in oddball trials, respectively, in the PM-task-repetition condition than in the ongoing-task-only condition. Negative beta weights signify smaller values in PM_REPEATED_ than in oddball trials, respectively, in the PM-task-repetition condition than in the ongoing-task-only condition. The positive interaction in the analysis of speed in the second third of a trial suggests inverse aftereffects in this segment. However, note, this interaction does not result in a significant main effect of the factor trial type in this segment. The negative interaction indicates increased aftereffects in the corresponding segment.PM = prospective memory

Our analysis of movement speed revealed a stronger response-slowing in the PM-task-repetition condition than in the ongoing-task-only condition over nearly the entire trial (time steps 1–100) and slower mouse movements in PM_REPEATED_ than in oddball trials during the last third of a trial (time steps 65–96) in both conditions. Most importantly, finished-phase condition and trial type interacted during two time segments. During the second third of a trial (time steps 35–47), aftereffects were smaller in the PM-task-repetition condition than in the ongoing-task-only condition. However, this effect was partly caused by an increased speed in PM_REPEATED_ trials compared to oddball trials in the PM-task-repetition condition. During the last quarter of a trial (time steps 81–93), aftereffects were more pronounced in the PM-task-repetition condition than in the ongoing-task-only condition.

**Fig. 5:**
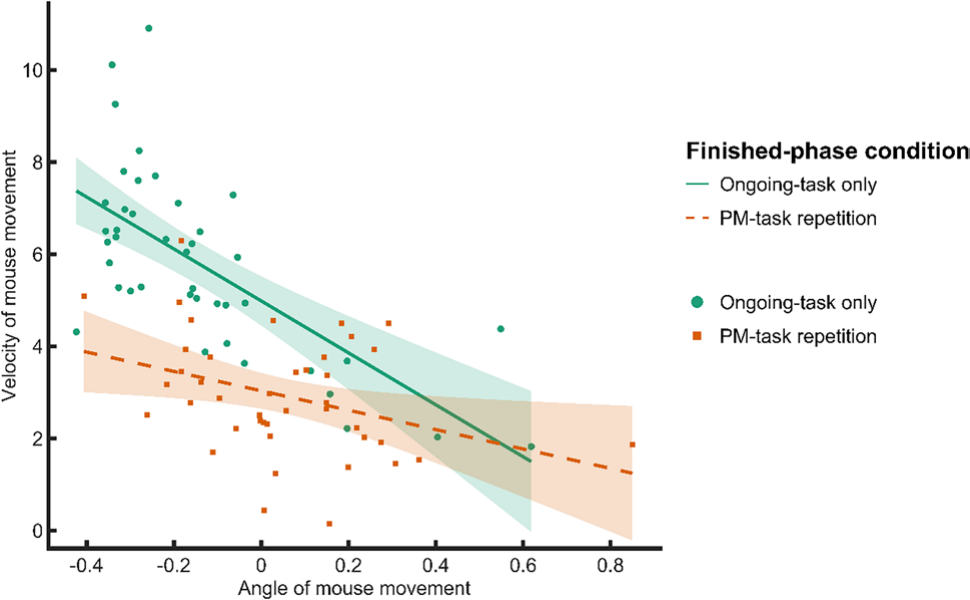
Correlation of the peak deviation of movement deflection in the second third of a trial and subsequent speed of mouse movement in the ongoing-task only condition (dashed) and the PM-task-repetition condition (solid) for each participant. PM = prospective memory

*Relation between movement deflection and slowing.* To test whether the extent of late response slowing in PM_REPEATED_ trials was contingent upon the extent of initial movement deflection, we assessed the relationship between these effects in an exploratory follow-up analysis. For this, we correlated each participants’ peak of movement deflection in the second third of a trial with the trough of speed during the last third (Fig. 5). In the ongoing-task-only condition, this analysis revealed a negative correlation between movement deflection and speed *r*(40) = -.68, *p* < .001, indicating that participants who initially showed a larger movement deflection toward the PM-response box subsequently also showed a slower speed. In the PM-task-repetition condition, this correlation was smaller *r*(40) = -.36, *p* = .02. Both correlations suggest that there may be a relationship between the deceleration of mouse movement and the previous movement deviation.

#### Costs to the ongoing task

To investigate whether costs to the ongoing task were caused by capacity-sharing monitoring processes or by a strategic delay, we compared mouse movements curvatures in standard trials between finished-phase conditions in a paired *t*-test. This analysis revealed that costs to the ongoing task were accompanied by greater curvature in finished phases of the PM-task-repetition condition (*M*_*z-score*_ = 0.05, *SD* = 0.1) than in the ongoing-task-only condition (*M*_*z-score*_ = -0.05, *SD* = 0.1), *t*(41) = 3.09, *p* = .004, *d* = 0.48, 95% CI [0.16, 0.79].

**Fig. 6:**
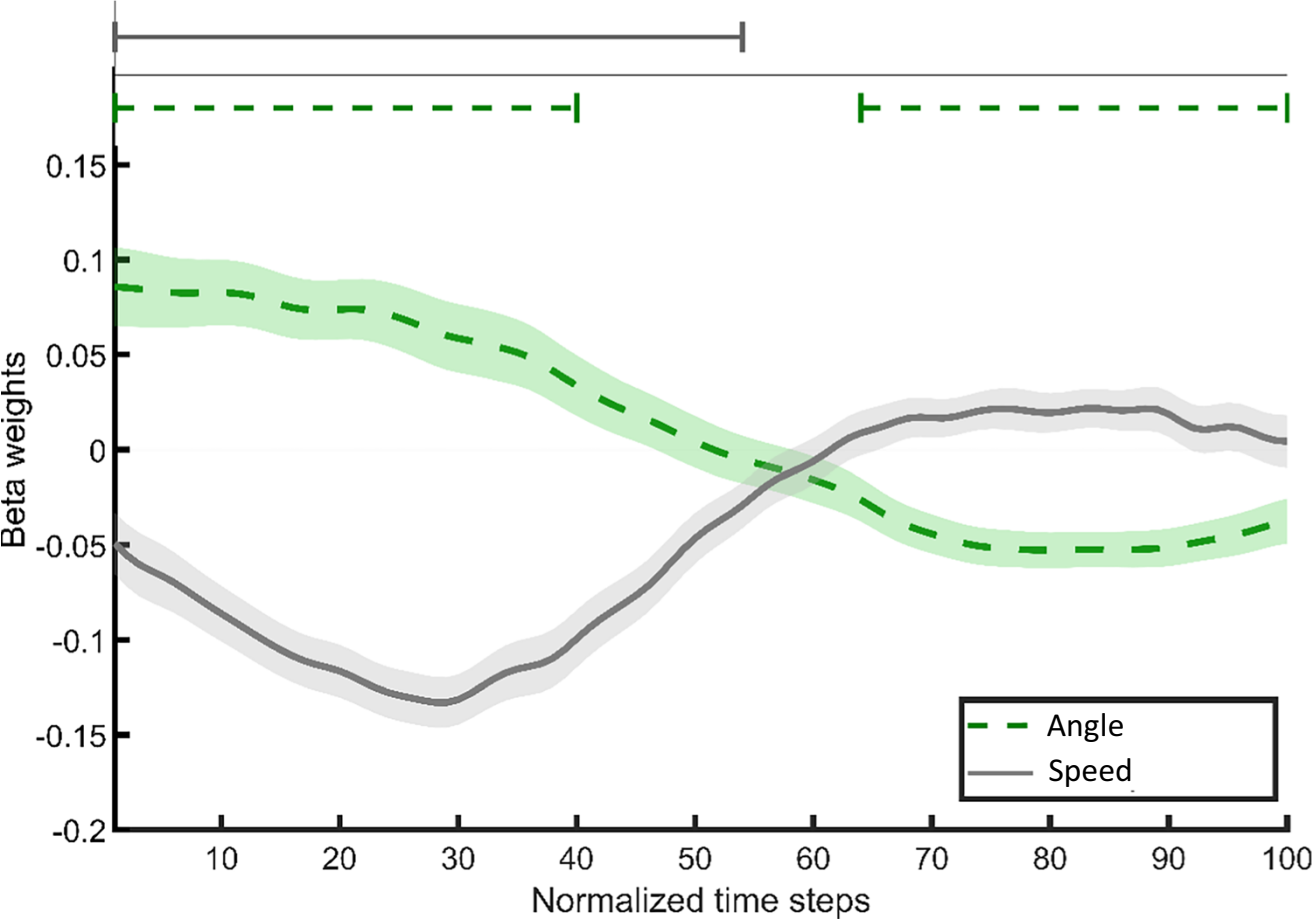
Results of continuous regression analyses on mouse movement in standard trials of the finished phase in the ongoing-task-only condition compared to the PM-task-repetition condition. The dashed line indicates the angle of the mouse movement. The solid line indicates the speed of the mouse movement. Positive beta-weights represent a stronger deflection of mouse movement in the direction of the PM-task response box (dashed line) or, respectively, a greater speed of mouse movement (solid line) in the PM-task-repetition condition than in the ongoing-task-only condition. Lines above the graphs indicate that segments of beta weights differ significantly from zero (*t*-test, a minimum of ten consecutive significant time steps). Confidence areas mark standard errors of beta weights

Subsequently, we performed continuous regression analyses on mouse-movement speed and angle in standard trials of finished phases with the finished-phase condition (PM-task-repetition, ongoing-task-only) as the predictor variable (Fig. 6). Consecutive significant time segments are shown in Table 3. Our analysis of movement speed revealed a stronger response slowing in the PM-task-repetition condition than in the ongoing-task-only condition during the first half of a trial (time steps 1–54), which suggests that the new PM task requires a time-consuming, cognitive process. Our analysis of movement angles revealed that mouse movement in the PM-task-repetition condition was initially more centered in the middle between the response boxes of the ongoing-task and PM task (time steps 1–40) than in the ongoing-task-only condition, which suggests that the new PM task causes a delay of responses. This centered movement was then followed by a stronger movement oriented toward the ongoing-task response boxes during the second half of a trial (time steps 64–100) in the PM-task-repetition condition compared to the ongoing-task-only condition.

**Table 3:** Consecutive significant time segments of the angle and the speed of mouse movement in time-continuous regression analyses with Finished-phase condition as the predictor variable

	Predictor variables	Movement angle	Movement speed
Consecutive significant time steps (*p* < .05)	PM-task-repetition condition vs. ongoing-task-only condition	[1, 40], positive [64, 100], negative	[1, 54], negative

*Note.* Numbers in brackets correspond to the start and endpoint time-series segments with consecutive significant beta weights in continuous regression analysis. In these segments, mouse-movement characteristics differed significantly between PM-task-repetition and ongoing-task-only conditions ([start segment, end segment]). The positive/negative characteristics describe the direction of these differences. Positive differences signify larger value in the PM-task-repetition condition than in the ongoing-only condition. Negative differences signify smaller values in the PM-task-repetition condition than in the ongoing-task-only condition. PM = prospective memory

## Discussion

In the present study, we aimed to clarify the role of the sub-processes of spontaneous intention retrieval for aftereffects of completed intentions and to investigate whether costs to the ongoing task while pursuing an event-based PM intention are caused by capacity sharing processes or a strategic response delay. To these aims, we developed a mouse-tracking paradigm that allowed us to distinguish the sub-processes of spontaneous intention retrieval as well as processes underlying costs to the ongoing task by assessing curvature, angle, and speed of mouse movements.

With the discrete response measures in our paradigm, we replicated previous findings of aftereffects of completed intentions in terms of slower ongoing-task responses in PM_REPEATED_ trials than in oddball trials, relatively few commission errors, and increased aftereffects when participants performed a novel PM task after intention completion (Anderson & Einstein, 2017; Walser et al., 2012; Walser et al., 2017). Additionally, we observed costs to ongoing-task performance in terms of slower ongoing-task responses when participants performed an event-based PM task than when they performed only an ongoing task (Anderson et al., 2019). Most importantly, our analyses of continuous response measures revealed differential effects in curvature, angle, and speed of mouse movements that we will now discuss in detail.

### Spontaneous retrieval of completed intentions

Our analysis of continuous mouse-movement measures revealed that mouse movements in PM_REPEATED_ trials exhibited a greater curvature toward the no-longer-relevant PM-response box than in oddball trials. However, as indicated by our time-continuous analysis of movement angles, this effect did not extend throughout the whole trial. Instead, it was strongest after approximately half of the movement. This movement deflection was then followed by a transient reduction of movement speed during the last quarter of the response when the initial movement deflection had already been corrected.

While a discrepancy-plus-search-based intention retrieval should have only decreased movement speed in PM_REPEATED_ trials, a reflexive-associative intention retrieval should have only increased movement curvature and angle in PM_REPEATED_ trials. Consequently, our analysis of movement deflection (curvature and angle) provides evidence for a reflexive-associative process involved in aftereffects of completed intentions. The late slowing of the ongoing-task response after movement correction is predicted neither by the discrepancy-plus-search process nor by the reflexive-associative process. While the discrepancy-plus-search process predicts a slowing of the ongoing-task response, it does so before the response is retrieved, and therefore, before the motion deviation that we found occurs.

We argue that the initial movement deflection suggests that aftereffects occur in response to spontaneously triggered reflexive-associative retrieval of the completed intention and/or no-longer-relevant PM response (Möschl et al., 2020). The small number of commission errors in our study and our finding that movement trajectories get back on track toward the ongoing-task response boxes corroborate the notion that intention deactivation and the prevention of commission errors, in particular, involves mobilization of cognitive control (Bugg et al., 2016; Bugg & Scullin, 2013; Schaper & Grundgeiger, 2019).

Our results have further implications for the dual-mechanisms account of intention deactivation. While this account states the involvement of cognitive control in intention deactivation and aftereffects, it does not (yet) consider whether an intention is retrieved by a reflexive-associative process or a discrepancy-plus-search process. In our view, the two retrieval processes imply different roles for cognitive control. On the one hand, cognitive control could be effective on the memory level, inhibiting the memory search in the discrepancy-plus-search process. Similarly, previous studies have hypothesized that aftereffects of completed intentions could result from a partially inhibited memory search (Anderson & Einstein, 2017). On the other hand, cognitive control could affect the response level, inhibiting the action component of a spontaneously retrieved intention (Bugg et al., 2016). In our view, the reflexive-associative account allows only operating of cognitive control at the response level because reflexive retrieval of an intention defines this process. Accordingly, the intention can only be inhibited after the intended action has been retrieved. Our results suggest that intention deactivation involves cognitive control by inhibiting a reflexively triggered PM response rather than an inhibited memory search. The movement deflection in PM _REPEATED_ trials suggests that the intended action has been retrieved from memory. In contrast, a successfully inhibited memory search would have impeded this movement deflection.

The late onset of the response slowing, after the movement had already returned to its normal state, has two critical implications. First, it suggests that the initial movement deflection in PM_REPEATED_ trials most likely does not result from an early discrepancy-plus-search process that is solved wrongly and leads to retrieving the no-longer-relevant PM response. If this had been the case, the associated movement slowing in a PM_REPEATED_ trial should have started before the movement deviation. Second, it seems unlikely to reflect discrepancy processing of the PM_REPEATED_ cue itself. Instead, we argue that the late-onset response slowing reflects a response verification process. In line with Schaper and Grundgeiger (2019), we assume that spontaneously retrieving the no-longer-relevant PM response and exerting control over intention execution to prevent making a commission error in a PM_REPEATED_ trial elicits an experience of a discrepancy, which then triggers a search in memory to verify whether interrupting the PM response was correct or not. This notion is further corroborated by preliminary evidence of a negative correlation between initial movement deflection and the peak of response slowing in our exploratory analysis, which suggests that the verification process seems to be more time-consuming the further the no-longer-relevant PM response had been performed initially. However, this correlation should be interpreted with caution because our sample size is too small to detect reliable correlations (Schönbrodt & Perugini, 2013). Furthermore, this result should be interpreted with caution, as other explanations for the late-trial slowing are possible. For example, it could indicate altered motor control processes. Future studies may investigate the relationship between movement deflection and deceleration in more detail by more appropriate measures.

### Costs to the ongoing task

Similar to our findings of ongoing-task performance costs in our discrete performance measures, our analysis of continuous response measures revealed altered mouse movements in the PM-task-repetition condition compared to the ongoing-task-only condition. Specifically, in the first half of a trial, mouse movements in standard trials were slower in the PM-task-repetition condition than in the ongoing-task-only condition. While this slowing could indicate a strategic delay and capacity sharing between the ongoing and PM task, our analyses of movement directions provide clear evidence for a strategic delay of ongoing-task responses. Specifically, we found that the slowing of mouse movement in standard trials during the PM-task-repetition condition was accompanied by a greater curvature and mouse movements that were more oriented toward the middle of the screen between ongoing-task and PM-task response boxes during the first half of a trial than in the ongoing-task-only condition. This movement pattern clearly suggests that participants delayed their decision in the ongoing task. A sharing of limited attentional resources between the ongoing and PM task (Einstein & McDaniel, 2010; Guynn, 2003; Smith, 2003) would not manifest in altered movement directions but should have only decreased movement speed in standard trials. By contrast, a strategic delay of ongoing-task responses (e.g., Heathcote et al., 2015) predicts such a movement in the PM-task-repetition condition. Consequently, we interpret the movement deviation as indicating a strategic delay. This interpretation is further corroborated by our finding of more careful responding (i.e., slower, but more accurate performance) in the PM-task-repetition condition, which is in line with predictions of delay theory but contrasts with capacity-sharing theories that predict no speed-accuracy tradeoff during PM tasks (Anderson et al., 2019).

Note that, although the direction measures in the present study provide evidence for a strategic delay of ongoing task responses, we cannot determine the extent to which the delay contributed to ongoing-task costs in the present study. Since there is evidence in research for capacity-sharing and strategic delay in PM tasks, it is reasonable to assume that both processes contribute to costs to the ongoing task (Anderson et al., 2019). Future studies may use altered study designs to make more precise statements on the composition of costs to the ongoing task.

### Effects of a new PM task on aftereffects of completed intentions

In contrast to our analysis of discrete effects that showed increased aftereffects when performing a novel PM task after intention completion, our analysis of movement data did not show overall increased aftereffects in the PM-task-repetition condition. Instead, we found that movement curvature in the PM-task-repetition condition was increased for PM_REPEATED_ and oddball trials resulting from a stronger orientation of movement angles toward the PM-response box during the first two thirds of responses in both types of trials. In addition, these movement deflections were accompanied by a more substantial overall response slowing throughout PM_REPEATED_ and oddball trials in the PM-task-repetition condition and a marked response slowing during the last third of PM_REPEATED_ trials in both conditions.

Interestingly, during the last third of a trial, movements were slower in PM_REPEATED_ than in oddball trials in both conditions. Furthermore, this effect was more pronounced in the PM-task-repetition than in the ongoing-task-only condition. Thus, the more substantial initial movement deflection toward the PM-response box and the more pronounced response slowing in both PM_REPEATED_ and oddball trials in the PM-task-repetition condition suggest that the requirement to perform another event-based PM task after intention completion generally increases the readiness to perform the PM response and at the same time increases response uncertainty during the processing of deviant stimuli. Additionally, the more pronounced late-onset movement slowing during PM_REPEATED_ trials in the PM-task-repetition condition suggests that performing a novel PM task after intention completion also prolongs the late-onset response verification process during PM_REPEATED_ trials after intention completion when the current task context still requires to occasionally perform the PM response albeit to a different PM cue.

In line with our previous findings (Möschl et al., 2020; Walser et al., 2017), we argue that the requirement to continuously perform PM tasks establishes a general PM-task set or retrieval mode (Guynn, 2003; Underwood et al., 2015) that increases participants’ sensitivity for detecting deviant stimuli. Consequently, processing any deviant stimuli (i.e., PM_REPEATED_ cues as well as oddballs) leads to a reflexive execution of the PM response, which in our study was often terminated successfully but was clearly visible in terms of a stronger movement deflection toward the PM-response box in PM_REPEATED_ and oddball trials. Additionally, we argue that this retrieval mode is associated with the strategic delay of ongoing-task responses that participants establish for a new PM task also increases the probability that PM_REPEATED_ cues are processed. This process, in turn, fosters reflexive retrieval of the completed intention and exacerbates aftereffects. Lastly, a general PM-task set or retrieval mode could also increase the probability of source-monitoring errors (Ball et al., 2011) or increase uncertainty about the source of the discrepancy experienced during PM_REPEATED_ trials, which could explain the more pronounced late-onset movement slowing during PM_REPEATED_ trials in the PM-task-repetition condition. This effect may also account for findings of commission errors with a novel PM response after intention completion (Walser et al., 2017) as well as findings of more frequent thoughts about the completed PM task following PM_REPEATED_ trials when participants performed a novel PM task after intention completion (Anderson & Einstein, 2017).

Although it was not the main focus of our analyses, we found preliminary evidence for somewhat privileged processing of PM_REPEATED_ compared to oddball cues when a new PM task is active. Descriptively, PM_REPEATED_ trials lead to a faster initial movement deflection than oddball trials in the PM-task-repetition condition. In the ongoing-task-only condition, we observed the opposite. Although neither effect reached significance on its own, the interaction analysis on speed revealed a significant effect. This was due to opposing effects in the PM-task-repetition and ongoing-task-only condition. Since we did not expect this effect, we can only speculate about its underlying processes. We interpret the interaction effect on speed to indicate the effects of a retrieval mode or a general PM-task set. We argue that the preparatory processes established by the retrieval mode may enhance the detection and processing of all kind of deviant stimuli. However, if participants expect to process the PM cues of a new intention, PM_REPEATED_ cues compared to oddballs continue to undergo privileged processing even after intention deactivation.

In addition to enhancing the processing of PM cues, the new PM task could affect the reflexive-associative process per se. Concerning this effect, a relatively older concept of prospective memory may influence the reflexive-associative retrieval of a completed intention when a new PM task is active – the intention-superiority effect (Goschke & Kuhl, 1993). This concept states that unfulfilled intentions have relatively higher subthreshold activation in memory compared to other memory contents. Based on this, the action-superiority effect specifies that the motor response component of an intention exhibits such increased subthreshold activation, making the PM response more readily accessible from memory after a PM cue is perceived (Freeman & Ellis, 2003). Freeman and Ellis (2003) hypothesized that the action-superiority effect could interact with spontaneous retrieval processes. In our study, the action superiority of a new intention might have fostered reflexive-associative retrieval processes, leading to the faster initiation of the corresponding action. Both processes may have resulted in the significant interaction of condition and trial type found in the second third of a trial regarding mouse movement speed. However, given the preliminary nature of this finding and main effects that did not reach significance in the corresponding section of a trial, future studies are required to assess the underlying effects in more detail.

Future studies are also needed to test whether the interaction effects we found are attenuated when completed, and novel PM tasks are more dissimilar, as has been observed previously (Walser et al., 2017). This could be a good starting point to disentangle further the various PM processes postulated in the literature on the level of perception, memory, and action.

### Limitations

A limitation of the present study regarding costs to the ongoing task is that we exclusively used non-focal cues in the PM task and, therefore, cannot provide any information on the mechanisms underlying costs to the ongoing task that may arise in PM tasks with focal PM cues. Previous studies have shown that costs to the ongoing task and the degree to which PM relies on spontaneous retrieval depend on the type of stimuli used in the PM task (e.g., focal vs. non-focal PM cues; McDaniel et al., 2015). In the present study, we have chosen non-focal cues to assess both spontaneous retrieval of completed intentions and ongoing-task costs within the same paradigm. Nevertheless, future studies may focus on the influence of the kind of stimuli used in the PM task to systematically investigate the degree to which the processes we observed in the present study are modulated by the involvement of spontaneous retrieval in PM.

The laboratory nature of the present study is another limitation to which the experimental study of PM is often subject. According to the multiphasic model (Kliegel et al., 2002; Scullin et al., 2013), PM involves the following phases: forming an intention, maintaining the intention, initiating the intended action, and executing the intention. Compared to real life, the maintaining phase in the laboratory is often relatively short in PM studies. This constraint was also the case in our study. After only a few ongoing-task trials (< 10), participants were confronted with the first PM trial. Therefore, we cannot rule out that a more prolonged maintaining phase would have affected our findings. In particular, the short maintaining interval may have resulted in participants not yet having sufficient time to fully deactivate a completed intention, which in turn would lead to an overestimation of the aftereffects of completed intentions in the present study. However, we consider this to be a minor limitation since, in this study, we were particularly interested in the detailed investigation of the processes that can lead to aftereffects and commission errors.

Also, the repetitive character of the paradigm is a possible confounding aspect. It is possible that the participants were confused by the change of different task conditions. However, everyday life often challenges us to complete an intention, deactivate it, form a new task to complete it, deactivate it, start another one, and so on. For this reason, we also consider this point as a minor limitation.

## Conclusion

The present study contributes to the growing body of research that found evidence for spontaneous retrieval of intentions. Using mouse tracking, we were able to shed light on the roles of reflexive-associative and discrepancy-plus-search processes for spontaneous retrieval of completed intentions. Our findings suggest that both processes contribute to aftereffects of completed intentions at different stages. First, a reflexive association causes a partial execution of the no-longer-relevant PM response. This execution goes along with the experience of a discrepancy, which then triggers a search process in memory regarding whether giving a PM response would be correct or not, in other words, whether the PM task is already completed. Finally, during the pursuit of another PM task, aftereffects of a previously completed PM task are exacerbated by stronger orienting responses to deviant stimuli during initial response phases as well as a prolonged memory search regarding the source of the experienced discrepancy during PM_REPEATED_ trials.

The present study also contributes to the current debate between capacity-sharing theories and the delay theory about why having an intention in mind slows ongoing activities. We found evidence that a strategic delay that could support the completion of an intention contributes significantly to this phenomenon. However, we could not rule out that the processes of capacity-sharing also contribute to this slowing.

Third, we were able to shed light on the interaction of processes of spontaneous retrieval of completed intentions and processes underlying costs to the ongoing task. In this regard, we argue that the strategic delaying of ongoing activities in order to improve PM comes at the cost of more pronounced aftereffects of completed intentions.

## Data Availability

The data and materials for all experiments are available at https://osf.io/zf78y/ .The experiment was not preregistered.

## Appendix

Symbols that served as PM cues, PM_REPEATED_ cues, and oddballs

## Footnotes

1 Note that previous studies (Boywitt & Rummel, 2012; Horn et al., 2011; Horn & Bayen, 2015) also used evidence-accumulation models to infer processes underlying PM and ongoing-task costs but used different terminology.

2 Note that we use the term spontaneous retrieval even when referring to situations where a new PM task is active. In this case, we use the term to refer to the processes subsumed under the term in PM research (i.e., discrepancy-plus-search and reflexive-association), although due to the new PM task processes related to PM are active.

3 Note that due to constraints of the mouse-tracking task, several task features in the present study deviated from the original experiment. For instance, number of trials and number of sessions in which subjects participated. Further, we replaced all non-symmetric symbols used by Walser et al. (2017) with symmetric ones. Most importantly, while in the study by Walser et al. (2017) ongoing tasks changed between active and finished phases, in the present experiment participants categorized digits as ongoing task during the entire experiment.
